# Sleep spindle deficits in antipsychotic-naïve early course schizophrenia and in non-psychotic first-degree relatives

**DOI:** 10.3389/fnhum.2014.00762

**Published:** 2014-10-07

**Authors:** Dara S. Manoach, Charmaine Demanuele, Erin J. Wamsley, Mark Vangel, Debra M. Montrose, Jean Miewald, David Kupfer, Daniel Buysse, Robert Stickgold, Matcheri S. Keshavan

**Affiliations:** ^1^Department of Psychiatry, Massachusetts General HospitalCharlestown, MA, USA; ^2^Athinoula A. Martinos Center for Biomedical ImagingCharlestown, MA, USA; ^3^Harvard Medical SchoolBoston, MA, USA; ^4^Department of Psychiatry, Beth Israel Deaconess Medical CenterBoston, MA, USA; ^5^Department of Psychiatry, Western Psychiatric Institute and Clinic, University of Pittsburgh School of MedicinePittsburgh, PA, USA

**Keywords:** sleep, sleep spindles, schizophrenia, cognition, IQ, polysomnography, endophenotype, relatives

## Abstract

**Introduction:** Chronic medicated patients with schizophrenia have marked reductions in sleep spindle activity and a correlated deficit in sleep-dependent memory consolidation. Using archival data, we investigated whether antipsychotic-naïve early course patients with schizophrenia and young non-psychotic first-degree relatives of patients with schizophrenia also show reduced sleep spindle activity and whether spindle activity correlates with cognitive function and symptoms.

**Method:** Sleep spindles during Stage 2 sleep were compared in antipsychotic-naïve adults newly diagnosed with psychosis, young non-psychotic first-degree relatives of schizophrenia patients and two samples of healthy controls matched to the patients and relatives. The relations of spindle parameters with cognitive measures and symptom ratings were examined.

**Results:** Early course schizophrenia patients showed significantly reduced spindle activity relative to healthy controls and to early course patients with other psychotic disorders. Relatives of schizophrenia patients also showed reduced spindle activity compared with controls. Reduced spindle activity correlated with measures of executive function in early course patients, positive symptoms in schizophrenia and IQ estimates across groups.

**Conclusions:** Like chronic medicated schizophrenia patients, antipsychotic-naïve early course schizophrenia patients and young non-psychotic relatives of individuals with schizophrenia have reduced sleep spindle activity. These findings indicate that the spindle deficit is not an antipsychotic side-effect or a general feature of psychosis. Instead, the spindle deficit may predate the onset of schizophrenia, persist throughout its course and be an endophenotype that contributes to cognitive dysfunction.

## Introduction

Sleep disturbances in schizophrenia have been described since Kraepelin (1919) and are common throughout its course (Lieberman et al., 2005), including in the prodrome (Miller et al., 2003). The presence of sleep disturbances in antipsychotic-naïve and unmedicated patients indicate that they are not merely a side-effect of medications (for meta-analysis see Chouinard et al., 2004). While often viewed as secondary to schizophrenia, as the accompanying psychological distress may itself diminish sleep quality (Benca, 1996), sleep deprivation can precipitate psychosis in vulnerable individuals (Tyler, 1955; Wright, 1993; but see, Kahn-Greene et al., 2007), and there is growing evidence that sleep disturbances can trigger or aggravate a range of psychiatric conditions (Wehr et al., 1987; Ford and Kamerow, 1989; Breslau et al., 1996; Turek, 2005; Huang et al., 2007; Germain et al., 2008; Sateia, 2009). In schizophrenia, sleep disturbances are seen in high-risk samples (Keshavan et al., 2004; Lunsford-Avery et al., 2013), are anecdotally associated with the initial onset of psychosis and predict psychotic decompensation in remitted patients (Dencker et al., 1986; Benson, 2006). If specific sleep abnormalities that contribute to the initial onset, relapse and manifestations of schizophrenia can be identified, they may serve as targets for intervention to prevent the emergence of schizophrenia, remediate its course and ameliorate core features.

Recent studies have reported that chronic, medicated patients with schizophrenia show a deficit in sleep spindles (Ferrarelli et al., 2007, 2010; Manoach et al., 2010; Seeck-Hirschner et al., 2011; Wamsley et al., 2012), which are a defining feature of non-rapid eye movement (NREM) Stage 2 sleep that are seen on the electroencephalogram (EEG) as brief (~1 s) bursts of synchronous activity in the 12–15 Hz range. This sleep spindle deficit occurred in the context of normal sleep architecture and Stage 2 spectral power, except in the sigma frequency band, which corresponds to the frequency range of sleep spindles. Here, we analyzed archival sleep data to determine whether young individuals at high genetic risk for schizophrenia (Keshavan et al., 2004) and antipsychotic-naïve early course patients with schizophrenia (Keshavan et al., 2011) have reduced sleep spindles and whether sleep spindle activity is related to cognitive function.

Animal studies point to sleep spindles as a key mechanism of synaptic plasticity, which may mediate memory consolidation during sleep (Rosanova and Ulrich, 2005; Werk et al., 2005). In humans, sleep spindles correlate with measures of intelligence and with sleep-dependent consolidation of both procedural and declarative memory (for review see, Fogel and Smith, 2011). In antipsychotic-naïve patients with schizophrenia, spindle activity is inversely related to reaction time on tests of attention (Forest et al., 2007). In chronic medicated patients with schizophrenia, reduced spindle activity predicts poorer recognition memory for words that were learned prior to sleep (Goder et al., 2008), impaired sleep-dependent motor procedural memory consolidation (Wamsley et al., 2012) and increased severity of positive symptoms (Ferrarelli et al., 2010; Wamsley et al., 2012). In a randomized placebo-controlled trial, chronic, medicated patients with schizophrenia were treated with eszopiclone (Lunesta®, a non-benzodiazepine hypnotic agent that acts on γ-aminobutyric acid (GABA) neurons in the thalamic reticular nucleus (TRN) where spindles are generated) showed a significant increase in spindle number, density and Stage 2 sigma power (Wamsley et al., 2013). These findings suggest that the spindle deficit in schizophrenia is a specific and treatable sleep abnormality that is related to cognitive dysfunction and symptoms.

Prior reports of decreased sleep spindles in chronic medicated patients with schizophrenia leave a number of important questions unresolved. For example, it is not known whether the spindle deficit is related to the pathophysiology of schizophrenia or to treatment with antipsychotic drugs. One study found that only antipsychotic-treated patients with schizophrenia, but not those with other psychotic disorders, showed deficient spindle activity (Ferrarelli et al., 2010) suggesting that the spindle deficit is neither an antipsychotic side-effect nor a general feature of psychosis. In contrast, several studies of unmedicated schizophrenia patients did not find evidence of a spindle deficit: Two studies reported normal spindle density during Stage 2 sleep in 11 (Poulin et al., 2003) and eight (Forest et al., 2007) antipsychotic-naïve patients; another reported normal spindle density in six unmedicated patients (Van Cauter et al., 1991); and one reported increased spindle density in five unmedicated patients (Hiatt et al., 1985). The latter two studies analyzed only selected NREM sleep segments and neither study distinguished between Stage 2 and slow wave sleep, making them difficult to compare with studies measuring spindle activity during all of Stage 2 sleep. Another unresolved question is whether spindle deficits are present in first-degree relatives. To address these questions we examined sleep spindles in young first-degree relatives of patients with schizophrenia and in antipsychotic-naïve patients recently diagnosed with psychosis. Sigma power (12–15 Hz), which is the frequency band of spindles, shows high heritability in twin studies, high inter-individual variability and within-individual stability over time suggesting that it is a genetically-mediated trait (Ambrosius et al., 2008; De Gennaro et al., 2008). The presence of spindle deficits in first degree relatives, early course and chronic schizophrenia patients would suggest that it is an endophenotype of schizophrenia. Endophenotypes are heritable traits that indicate genetic vulnerability to illness (Gottesman and Gould, 2003). They are associated with illness but are also present in some syndromally-unaffected relatives.

We also investigated the association of sleep spindles with cognitive performance, functional assessments and symptom ratings. Since sleep spindles positively correlate with performance on a range of cognitive measures in both healthy individuals (Fogel and Smith, 2011) and patients with schizophrenia (Forest et al., 2007; Goder et al., 2008; Seeck-Hirschner et al., 2011; Wamsley et al., 2012), we expected to observe similar relations in our experimental and control samples. Based on prior findings, we also expected reduced spindle activity to correlate with positive symptoms in schizophrenia (Ferrarelli et al., 2010; Wamsley et al., 2012).

## Methods

### Participants

Demographic and descriptive data are given in Table 1. For all samples, potential participants were excluded if they met DSM-IV criteria (American Psychiatric Association, 2000) for current substance abuse or dependence.

**Table 1:** **Demographic characteristics and description of study samples**.

	**Patients *n* = 26**			**Controls *n* = 25**	***p***	**Relatives *n* = 19**	**Controls *n* = 12**	***p***
	**SZ *n* = 15**		**Other *n* = 11**					
Age		27 ± 7		27 ± 7	0.96	14 ± 4	15 ± 5	0.61
	28 ± 8		27 ± 7		0.74			
Sex (#/% M)^a^		17/65		16/64	0.92	9/47	7/58	0.82
	11/73		6/55		0.56			
Education (years)		14 ± 2		16 ± 2	<0.001^*^	8 ± 4	9 ± 4	0.63
	13 ± 2		14 ± 2		0.04^*^			
Est. IQ		99 ± 13				96 ± 12	110 ± 16	0.01^*^
	97 ± 10		101 ± 17		0.43			
Parental SES						29 ± 11	52 ± 10	<0.001^*^

*Means ± SD, SZ, schizophrenia; M, male; SES, socio-economic status. p-values are based on t-tests*.

* *Significant at p ≤ 0.05*.

a *p-values are based on chi-square tests*.

#### Early course participants and controls

Twenty-six inpatients and outpatients were recruited from the Western Psychiatric Institute and Clinic based on having a newly diagnosed psychotic disorder confirmed in consensus meetings led by senior clinicians (MSK, DM) using all clinical data including Structured Clinical Interviews for DSM-IV (SCID, First et al., 1997). Patients were diagnosed with schizophrenia (*n* = 15); major depression (*n* = 4); delusional disorder (*n* = 2); schizoaffective disorder (*n* = 2); bipolar disorder (*n* = 2); mood disorder, NOS (*n* = 1). Patients were characterized with the Scales for the Assessment of Positive and Negative Symptoms (SAPS and SANS, Andreasen, 1983, 1990) and the Global Assessment of Functioning Scale (GAF, American Psychiatric Association, 2000) within a week of the sleep studies. The following neuropsychological assessments were administered: Ammon's Quick Test, a pointing picture vocabulary test, to estimate verbal IQ (Otto and McMenemy, 1965); the Wisconsin Card Sort Test (WCST, Berg, 1948); Trail Making Tests Parts A and B (Reitan, 1958); the Block Design subtest of the Wechsler Adult Intelligence Scale-Revised (WAIS-R, Wechsler, 1981); the Wide Range Achievement Test-Revised, Reading portion (WRAT-R, Jastak and Wilkinson, 1984) and immediate recall of the California Verbal Learning Test (Delis et al., 1988). Supplemental Table 1 presents neuropsychological data.

The 15 early course patients diagnosed with schizophrenia were similar in age, sex, and estimated IQ to the 11 patients with other psychotic disorders, but had completed one less year of education, a difference that was statistically significant (Table 1). The early course groups did not differ significantly in ratings of positive or negative symptom severity or global functioning (GAF) (Supplemental Table 2). While they did not differ significantly on neuropsychological measures (Supplemental Table 1), with the exception of WRAT-R reading, which is often used to estimate pre-morbid verbal IQ, schizophrenia patients generally performed at a lower level. Two patients with schizophrenia and three patients with other psychotic disorders reported current cigarette use.

Sleep and cognitive data on these patients were presented in a prior publication (Keshavan et al., 2011) The present report includes a healthy control group for comparison, considers patients with schizophrenia separately from those with other psychoses and measures sleep spindles rather than sigma frequency power.

Twenty-five healthy individuals, screened to exclude a personal history of mental illness and present substance abuse (SCID-Non-patient edition; First et al., 2002), were recruited from the local community by advertisement, word of mouth and presentations to community groups. The healthy controls were matched to the patients for age and sex but had completed significantly more years of education. They were not administered neuropsychological assessments and no information on parental socioeconomic status or cigarette use was available.

#### First-degree relatives and their controls

A total of 19 children (*n* = 17) and siblings (*n* = 2) of patients with SCID confirmed diagnoses of schizophrenia were recruited by first asking the patient's permission to approach their relative. Relatives were included if they never had a psychotic disorder and were not taking antipsychotic drugs. Thirteen of the high-risk sample were diagnosed with a lifetime history of other disorders: Attention Deficit Disorder (*n* = 5); major depression (*n* = 2); separation anxiety disorder (*n* = 2); oppositional defiant disorder (*n* = 2); and conduct disorder (*n* = 2). One relative was taking amphetamine and dextroamphetamine (Adderall) and another was taking sertraline at the time of the sleep study.

Twelve age, sex and education matched healthy individuals without a personal history of mental illness or any first-degree family members with an Axis I disorder (confirmed by SCID interviews), were recruited from the community (as above) as control participants (Table 1). Control participants had a significantly higher IQ estimates and parental socioeconomic status (SES, Hollingshead, 1965) than the high-risk relatives. Two of the relatives and none of the control participants reported current cigarette use.

Relatives and controls were administered a SCID, SCID-Non patient version or, for children under 15, the children's epidemiological version of the Schedule for Affective Disorders and Schizophrenia (K-SADS-E, Orvaschel and Puig-Antich, 1987). Potential participants with substance abuse within 4 weeks of the initial assessment or alcohol dependence within the previous 2 years were excluded.

Relatives and controls were characterized with the Chapman Psychosis Proneness Scales of Magical Ideation (Eckblad and Chapman, 1983), Perceptual Aberration (Chapman et al., 1978), and Social Anhedonia (Mishlove and Chapman, 1985), the GAF, and the Premorbid Adjustment Scale (PAS, Cannon-Spoor et al., 1982). Relatives had significantly higher ratings of Magical Ideation and Perceptual Aberration and significantly lower GAF and PAS ratings (Supplemental Table 2). The following neuropsychological measures were administered to relatives and controls: the Ammon's Quick Test, the WCST, and the Continuous Performance Test—Identical Pairs version (CPT-IP, Cornblatt et al., 1988) (Supplemental Table 1).

#### Consent

Experimental protocols were approved by the University of Pittsburgh School of Medicine Institutional Review Board. All participants provided written informed consent (or assent if under 18) following a full description of the study. The parent or guardian also provided informed consent for participants younger than 18.

### Procedures

#### Polysomnography (PSG)

Sleep studies were conducted at the Western Psychiatric Institute and Clinic sleep lab over two consecutive nights. For several days prior to the sleep study, participants were asked to refrain from napping during the day. Sleep times were based on habitual “good night” and “good morning” times, determined using a participant diary of recent sleep patterns. PSG electrodes were placed approximately 1 h before bedtime. Sleep data were acquired at 128 Hz using Grass Telefactor M15 bipolar Neurodata amplifiers and locally-developed collection software. The recording montage consisted of bilateral central (C3 and C4) electroencephalogram (EEG) leads referenced to the linked mastoids (A1+A2); right and left electrooculogram (EOG) referenced to A1+A2; and bipolar submental chin electromyogram (EMG). We analyzed data from the second night. Each 30 s epoch of PSG data was visually classified into stages (Wake, NREM 1, 2, slow wave sleep, and REM) according to standard criteria (Rechtschaffen and Kales, 1968) by a rater blind to diagnostic group. The classified sleep data were segmented into 30 s segments for subsequent data analyses.

#### Sleep spindle analysis

As in our prior studies, we analyzed spindles during Stage 2 sleep (Manoach et al., 2010; Wamsley et al., 2012, 2013). PSG data were preprocessed and analyzed using BrainVision Analyzer (version 2.0.2, BrainProducts, Munich Germany) and MATLAB (version R2009b, The MathWorks, Natick MA) software. Prior to analysis, data were filtered at 0.3–35 Hz and artifacts were rejected by manual inspection. Discrete sleep spindle events were automatically detected at the C4 lead, which was the only lead available for all participants, using a wavelet-based algorithm that was previously validated against both hand-counted spindles and 12–15 Hz sigma power in both healthy individuals and patients with schizophrenia (Wamsley et al., 2012) and outperformed other available automated spindle detectors by most closely approximating expert consensus spindle counts (Warby et al., 2014).

For each spindle, measures of amplitude, sigma power, duration, and peak frequency were based on analysis of 2 s EEG epochs centered on the point of spindle detection. Within the sigma range (12–15 Hz), *amplitude* was the maximal voltage following 12–15 Hz band pass filtering, *peak frequency* was defined as the spectral peak of the spindle following Fast Fourier transform (FFT) decomposition, and *sigma power* was defined as the mean FFT-derived power spectral density in the 12–15 Hz range (μV^2^/Hz). To examine the time-frequency characteristics of individual spindles, wavelet analysis was conducted. A complex Morlet wavelet was applied separately to each spindle epoch. The *duration* of each spindle was calculated as the half-height width of wavelet energy within the spindle frequency range.

We chose spindle density (events/min) and individual spindle amplitude as our primary dependent variables for regressions with cognitive and symptom measures. Spindle density was chosen because it is more resistant to group differences in total sleep time (TST) than spindle number, was deficient in our prior studies of chronic medicated patients and correlated with sleep-dependent memory consolidation (Manoach et al., 2010; Wamsley et al., 2012). Spindle amplitude was chosen because it negatively correlated with positive symptoms in our prior study (Wamsley et al., 2012) and contributes to the measurement of “integrated spindle activity,” which negatively correlated with positive symptoms in a study from another group (Ferrarelli et al., 2010).

#### Spectral characterization of stage 2 sleep

The power spectral density (μV^2^/Hz) was calculated by FFT, using a Hanning window with 50% overlap applied to successive 3 s epochs of Stage 2 sleep. Spectral power in the slow oscillation (0.5–1 Hz), delta (1–4 Hz), theta (4–8 Hz), alpha (8–12 Hz), sigma band (12–15 Hz), and beta (15–30 Hz) frequency bands was measured.

#### Spindle density and amplitude in relation to cognition, function, and symptom ratings

Cognitive, function or symptom measurements were regressed on the primary spindle parameters (density and amplitude) using robust regression models, which limit the influence of outliers on the results (Andersen, 2008), as implemented in MATLAB. Group (*x*_2_) and its interaction with spindle parameter (*x*_1_) were included in the model: *y* = β_0_+β_1_*x*_1_+β_2_*x*_2_+β_3_*x*_1_*x*_2_. In the early course sample, group refers to schizophrenia patients vs. those with other psychotic disorders (for early course controls cognitive data were not available). In the high-risk sample, group refers to relatives vs. controls. If the group factor (difference in intercepts) and the group by spindle parameter interaction (difference in slopes) are not significant, we report the relations for the pooled group data without factors for group and its interaction with the sleep parameter (*y* = β_0*p*_+β_1*p*_*x*). Otherwise we also report standard linear regression results for each group separately.

## Results

### Early course participants (Table 2)

#### Sleep quality

Early course patients showed worse sleep quality than controls with significantly less TST, more wake time after sleep onset (WASO), and lower sleep efficiency (TST/total time in bed). Although both groups of early course patients showed disrupted sleep compared with controls, in schizophrenia patients the disruption tended to be worse as indexed by trends toward more WASO and lower sleep efficiency.

**Table 2:** **Sleep data for early course patients and their controls reported as means *±* SD**.

	**Patients *n* = 26**			**Controls *n* = 25**	**Patients vs controls**.		**Sz vs. controls**		**Sz vs. others**		**Others vs controls**.	
	**SZ *n* = 15**		**Other *n* = 11**		***t***	***p***	***t***	***p***	***t***	***p***	***t***	***p***
**SLEEP QUALITY**												
TST (min)		403 ± 76		443 ± 30	−2.51	0.02^*^	−1.86	0.07	0.62	0.54	−3.08	0.004^*^
	411 ± 80		392 ± 73									
WASO (min)		37 ± 39		15 ± 16	2.61	0.01^*^	3.79	<0.001^*^	1.93	0.07	0.69	0.49
	49 ± 40		21 ± 33									
Sleep efficiency %		85 ± 11		94 ± 4	−3.77	<0.001^*^	−4.68	<0.001^*^	−1.69	0.10	−2.23	0.03^*^
	81 ± 12		89 ± 9									
**SLEEP ARCHITECTURE**												
Stage 1 %		4 ± 2		4 ± 3	0.04	0.97	0.45	0.65	1.14	0.27	−0.48	0.63
	5 ± 2		4 ± 2									
Stage 2 %		62 ± 8		52 ± 9	4.41	<0.001^*^	4.52	<0.001^*^	1.19	0.25	2.56	0.02^*^
	64 ± 7		60 ± 9									
SWS %		7 ± 6		17 ± 7	−5.19	<0.001^*^	−5.15	<0.001^*^	−1.23	0.23	−3.03	0.005^*^
	6 ± 5		9 ± 7									
REM %		26 ± 5		27 ± 6	−0.54	0.59	−0.87	0.39	−0.88	0.39	0.14	0.89
	26 ± 6		28 ± 4									
**SPECTRAL POWER DURING STAGE 2 SLEEP (μV^2^/HZ)**												
Slow (0.5–1 Hz)		6.2 ± 2.5		8.4 ± 4.6	−2.12	0.04^*^	−1.79	0.08	−0.20	0.84	−1.37	0.18
	6.2 ± 2.2		6.3 ± 3.0									
Delta (1–4 Hz)		1.23 ± 0.37		1.55 ± 0.59	−2.29	0.03^*^	−2.38	0.02^*^	−1.33	0.20	−1.03	0.31
	1.15 ± 0.33		1.35 ± 0.41									
Theta (4–8 Hz)		0.16 ± 0.06		0.23 ± 0.10	−2.92	0.005^*^	−3.01	0.005^*^	−2.16	0.04^*^	−1.26	0.22
	0.14 ± 0.05		0.19 ± 0.05									
Alpha (8–12 Hz)		0.09 ± 0.05		0.12 ± 0.08	−1.43	0.16	−1.54	0.13	−1.11	0.28	−0.54	0.60
	0.08 ± 0.06		0.10 ± 0.04									
Sigma (12–15 Hz)		0.16 ± 0.1		0.22 ± 0.12	−1.82	0.08	−2.72	0.01^*^	−2.68	0.01^*^	−0.03	0.98
	0.12 ± 0.08		0.22 ± 0.09									
Beta (15–30 Hz)		0.007 ± 0.003		0.007 ± 0.004	−0.42	0.67	−0.40	0.69	−0.16	0.87	−0.25	0.81
	0.007 ± 0.004		0.007 ± 0.002									
**SPINDLE MEASURES**												
Spindle number		346 ± 124		374 ± 92	−0.92	0.36	−1.68	0.10	−1.56	0.13	0.44	0.66
	314 ± 133		389 ± 101									
Spindle density/min		1.41 ± 0.47		1.64 ± 0.32	−2.01	0.05^*^	−3.32	0.002^*^	−2.78	0.01^*^	0.32	0.75
	1.22 ± 0.48		1.67 ± 0.30									
**CHARACTERISTICS OF INDIVIDUAL SPINDLES**												
Amplitude (μV)		16.1 ± 4.1		18.0 ± 4.6	−1.62	0.11	−2.17	0.04^*^	−1.89	0.07	−0.19	0.85
	14.8 ± 4.4		17.7 ± 3.1									
Frequency (Hz)		13.1 ± 0.9		13.1 ± 0.9	−0.17	0.87	−0.44	0.66	−0.62	0.54	0.26	0.80
	13.0 ± 0.9		13.2 ± 0.8									
Duration (s)		0.813 ± 0.044		0.826 ± 0.044	−0.87	0.39	−1.74	0.09	−2.16	0.04^*^	0.65	0.52
	0.800 ± 0.047		0.835 ± 0.031									
Sigma power (μV^2^/Hz)		0.607 ± 0.917		0.519 ± 0.363	0.45	0.66	0.46	0.65	0.19	0.85	0.37	0.71
	0.637 ± 1.20		0.566 ± 0.315									

*TST, total sleep time; WASO, wake after sleep onset; SWS, slow wave sleep; REM, rapid eye movement sleep. Asterisks denote significance at p ≤ 0.05*.

#### Sleep architecture

Relative to controls, early course patients showed a greater percentage of Stage 2 sleep and a reduced percentage of slow wave sleep. This was true of both schizophrenia patients and those with other psychotic disorders.

#### Spectral characteristics of stage 2 sleep

Relative to controls, early course patients showed reduced slow oscillation, delta, and theta power. Of these, only the reduction in theta power significantly differentiated schizophrenia patients from those with other psychotic disorders.

**Figure 1:**
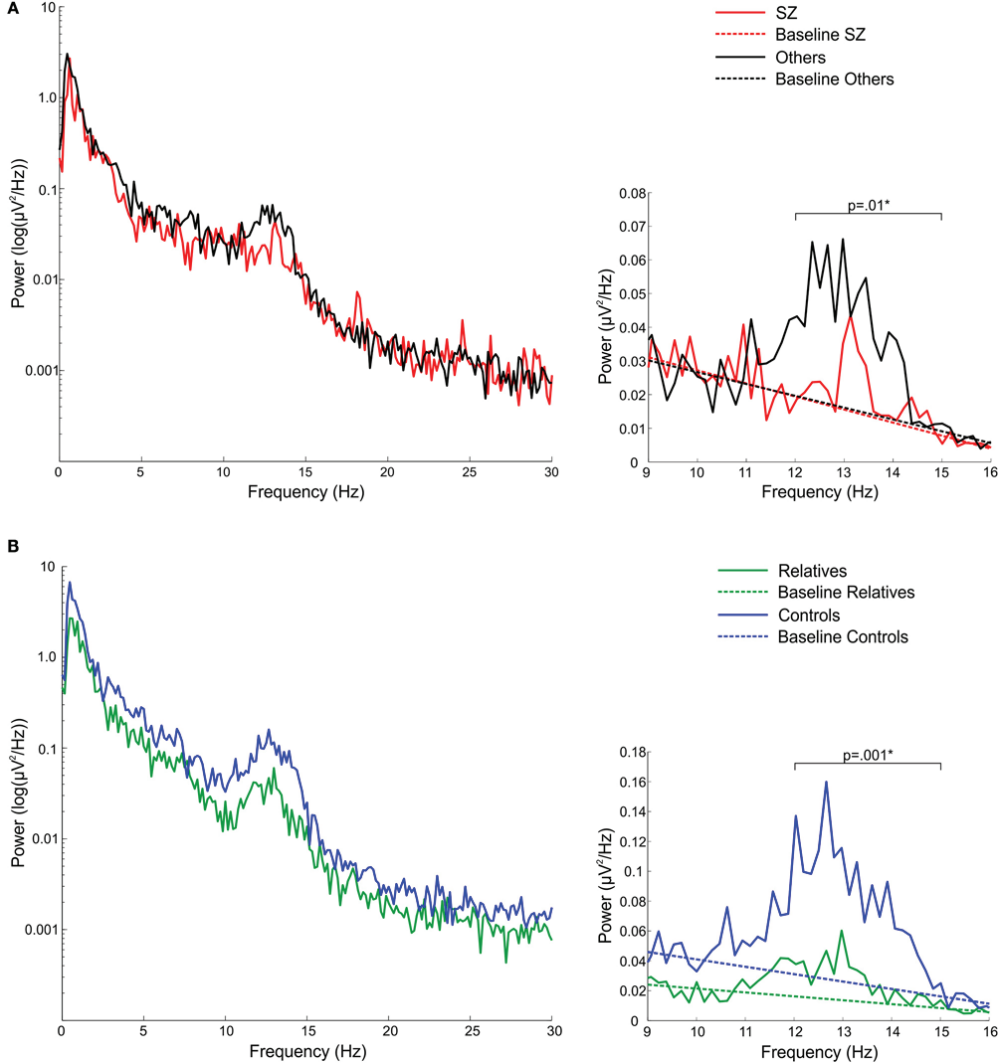
**Spectral power plots (A) in schizophrenia patients (SZ) and patients with other psychotic disorders (Others) and (B) in relatives and healthy controls**. The left plots show the logarithm of spectral power across the broader 0–30 Hz range. The right plots show 9–16 Hz spectral power with the sigma band (12–15 Hz) EEG power baseline for each group, which was computed as the best fit to the to the 9–10 and 15–16 Hz EEG power baseline. The *p*-values reflect group differences in sigma power. Asterisks denote significance at *p* = 0.05.

In the sigma frequency band, which corresponds to the frequency range of sleep spindles, schizophrenia patients showed significantly reduced sigma power compared with both other psychotic patients and controls. Psychotic patients with other disorders did not differ from controls in sigma power. So while both patient groups showed reduced spectral power in multiple frequency bands during Stage 2 sleep, only schizophrenia patients showed a sigma deficit. When calculated relative to the EEG power baseline for each group, computed as the best fit to the 9–10 and 15–16 Hz data, the sigma power (12–15 Hz) in schizophrenia patients was only 27% of that seen in patients with other psychoses (Figure 1A).

#### Sleep spindle parameters

**Figure 2:**
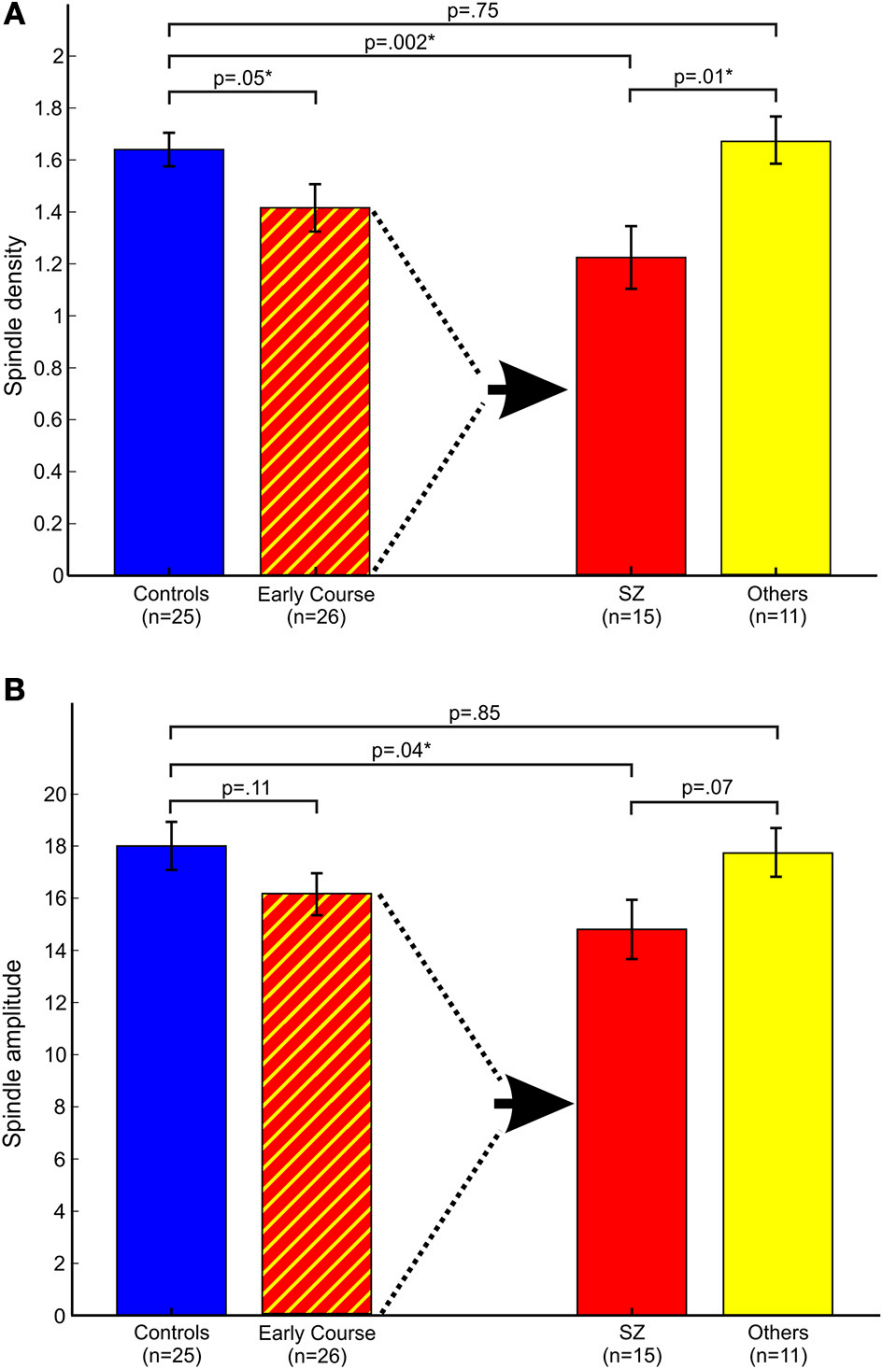
**Spindle parameters in controls and early course patients**. Early course patients are divided based on a diagnosis of schizophrenia (SZ) vs. other psychotic disorders (Others). **(A)** Spindle density; **(B)** Spindle amplitude. Asterisks denote significance at *p* = 0.05.

Relative to controls, early course patients showed significantly reduced spindle density (Figure 2A). This reduction was entirely due to the subset of patients diagnosed with schizophrenia who had significantly lower spindle density than both controls and patients with other psychotic disorders whose spindle density was nearly identical to that of controls. Schizophrenia patients also showed reduced spindle amplitude (Figure 2B) and duration compared with controls (trend for duration) and patients with other psychotic disorders (trend for amplitude). Patients with other psychotic disorders did not differ from controls on any spindle parameter.

#### Spindle density and amplitude in relation to cognition and symptom ratings (Table 3, Figure 3)

In the pooled group of early course patients, lower spindle density was associated with worse cognitive performance on all cognitive measures except immediate recall of the CVLT word list. Lower spindle density significantly predicted slower completion of Trails A and B, increased perseverative errors on the WCST, lower WRAT-R reading scores and lower estimated verbal IQ. Lower spindle density also predicted lower scaled scores on the Block Design subtest of the WAIS-R, but at a trend level. With the exception of estimated verbal IQ, these relations did not differ significantly as a function of group (schizophrenia, other psychotic disorders). Although the relation with IQ was in the same direction in both groups [significant in the non-schizophrenia psychotic patients: *t*_(9)_ = 2.29, *p* = 0.05; at a trend level in schizophrenia: *t*_(13)_ = 1.95, *p* = 0.07], the regression lines differed significantly (Table 3, Figure 4).

**Table 3:** **Regressions of cognitive and symptom measures on sleep parameters (spindle density, spindle amplitude, or sleep efficiency) in early course patients**.

		**Group**		**Sleep parameter× group**		**Sleep parameter (pooled data)**	
		***t***	***p***	***t***	***p***	***t***	***p***
Trails A (s)	Spindle density	−0.98	0.34	0.78	0.44	−2.02	0.05^*^
	Spindle amplitude	−1.34	0.19	1.28	0.21	−0.80	0.43
	Sleep efficiency	−0.54	0.59	0.62	0.54	0.03	0.98
Trails B (s)	Spindle density	0.54	0.60	−0.18	0.86	−5.36	<0.0001^*^
	Spindle amplitude	0.21	0.84	0.11	0.91	−5.22	<0.0001^*^
	Sleep efficiency	0.45	0.66	−0.19	0.84	−2.13	0.04^*^
WCST perseverative errors	Spindle density	−0.76	0.46	0.71	0.49	−2.49	0.02^*^
	Spindle amplitude	−1.95	0.06	2.02	0.06	−2.06	0.05^*^
	Sleep efficiency	1.28	0.22	−1.22	0.24	−4.70	0.0001^*^
IQ estimate	Spindle density	2.05	0.05^*^	−2.14	0.04^*^	2.96	0.007^*^
	Spindle amplitude	2.34	0.03^*^	−2.35	0.03^*^	2.29	0.03^*^
	Sleep efficiency	1.57	0.13	−1.58	0.13	1.39	0.18
WRAT-R	Spindle density	1.42	0.17	−1.31	0.21	3.19	0.004^*^
reading	Spindle amplitude	2.85	0.01^*^	−2.77	0.01^*^	2.09	0.05^*^
	Sleep efficiency	1.54	0.14	−1.52	0.14	1.51	0.14
Block design	Spindle density	−0.12	0.90	0.13	0.90	1.90	0.07
	Spindle amplitude	1.73	0.10	−1.71	0.10	3.12	0.005^*^
	Sleep efficiency	0.64	0.53	−0.69	0.51	2.37	0.03^*^
CVLT	Spindle density	−0.06	0.95	−0.20	0.84	0.27	0.79
	Spindle amplitude	0.07	0.94	−0.24	0.81	0.85	0.40
	Sleep efficiency	−0.46	0.65	0.40	0.69	1.84	0.08
SANS	Spindle density	0.89	0.38	−0.57	0.58	0.66	0.52
	Spindle amplitude	−1.27	0.22	1.49	0.15	−0.78	0.44
SAPS	Spindle density	0.99	0.33	−0.79	0.44	0.44	0.66
	Spindle amplitude	−0.81	0.42	1.10	0.28	1.18	0.25
GAF	Spindle density	−0.68	0.50	0.38	0.71	0.25	0.80
	Spindle amplitude	0.32	0.75	−0.60	0.56	0.40	0.69

*Group: schizophrenia vs. other psychotic disorders. The Group (x_2_) and Sleep parameter (x_1_) x Group columns are based on the following model: y = β_0_ + β_1_x_1_+ β_2_x_2_ + β_3_x_1_x_2_. The sleep parameter column is based on the regression using pooled group data without factors for group and its interaction with the sleep parameter (y = β_0p_ + β_1p_x). WCST, Wisconsin Card Sort Test; WRAT-R, Wide Range Achievement Test-Revised; Block Design scaled score; CVLT, California Verbal Learning Test standard score for total immediate word recall; SANS, Scale for the Assessment of Positive Symptoms global total; SAPS, Scales for the Assessment of Positive Symptoms global total; GAF, Global Assessment of Functioning*.

* *Significant at p ≤ 0.05*.

**Figure 3:**
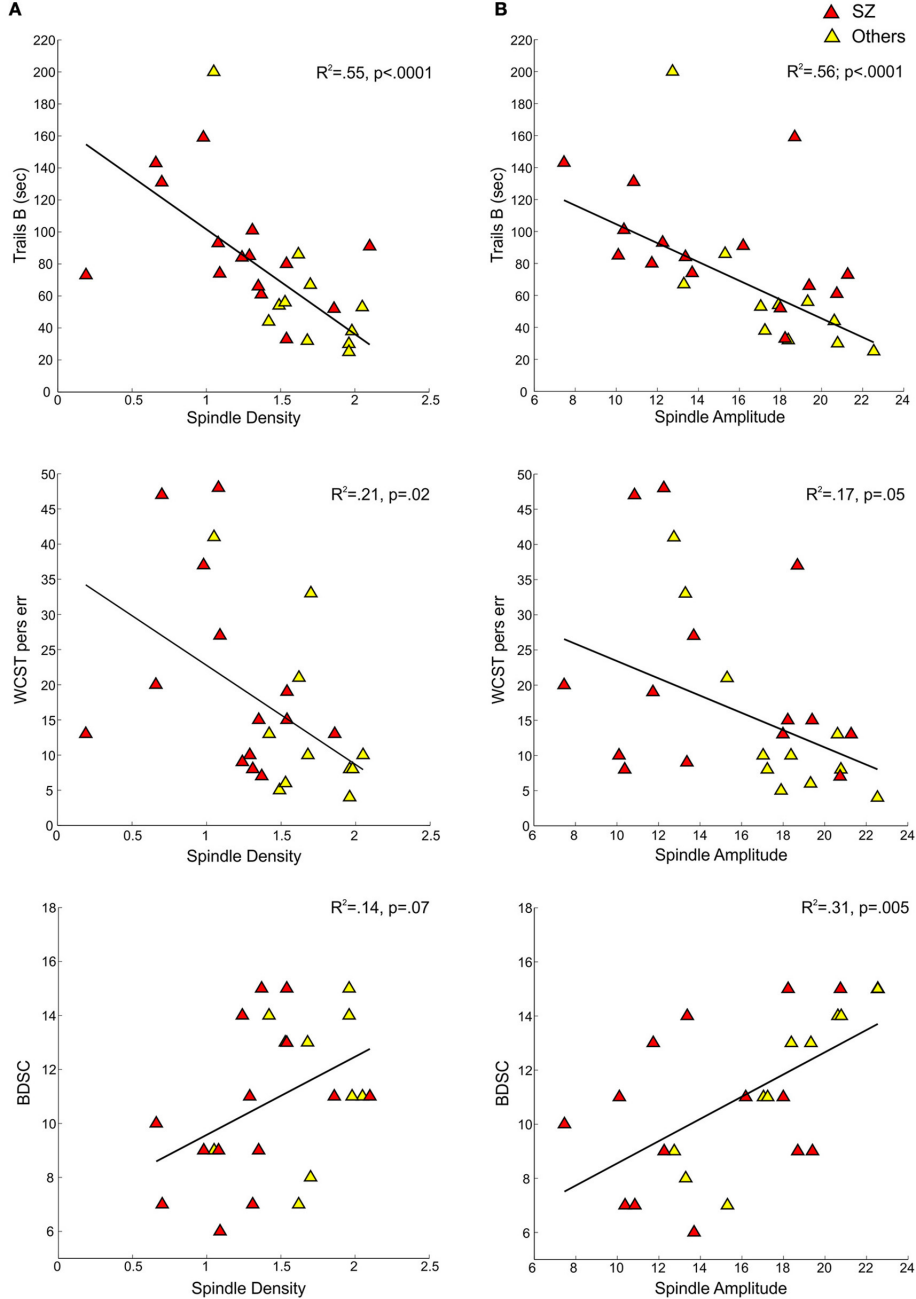
**Regressions of cognitive measures on spindle density and amplitude for early course schizophrenia patients (SZ) and those with other psychotic disorders (Others)**. **(A)** Shows cognitive measures—Trails B, WCST perseverative errors, and Block Design scaled score—regressed on spindle density. **(B)** Shows the same cognitive measures regressed on spindle amplitude.

**Figure 4:**
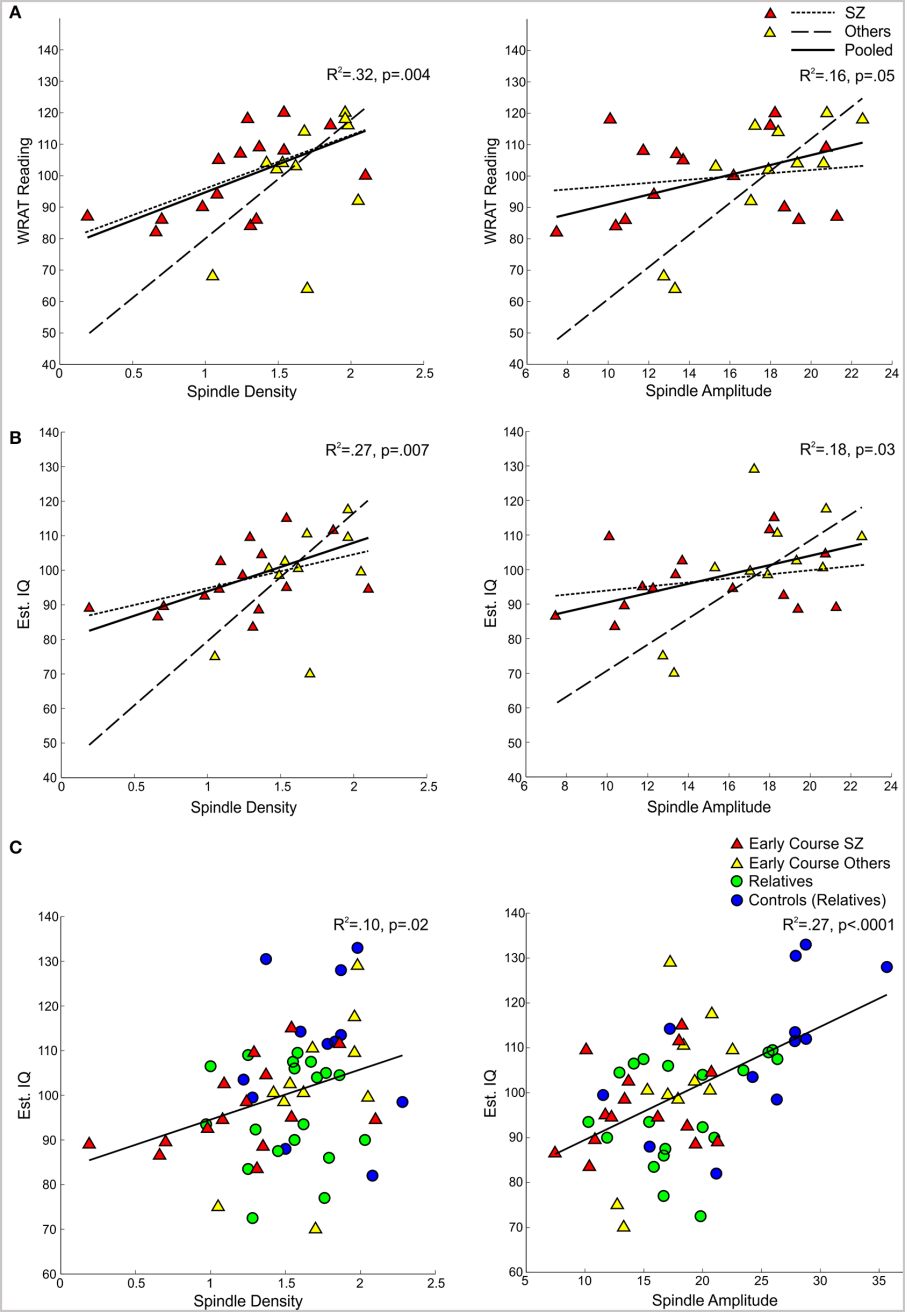
**Regressions of WRAT-R Reading standard scores and estimated verbal IQ from the Ammons Quick Test on spindle density and amplitude. (A,B)** Early course patients with schizophrenia (SZ) and other psychotic disorders (Others) with regression lines for each group and the pooled group data. **(C)** Regression of estimated verbal IQ on spindle density and amplitude for the pooled group data from early course schizophrenia, other early course psychotic patients, relatives, and relatives' controls.

Spindle amplitude also correlated with cognitive performance. Like spindle density, lower spindle amplitude was associated with slower performance on Trails B, increased WCST perseverative errors and a lower score on Block Design and these relations did not differ by group. Reduced spindle amplitude also correlated with lower estimated verbal IQ and WRAT-R reading scores in the pooled data, but there was an effect of group reflecting that only the non-schizophrenia psychotic patients showed significant relations of amplitude with estimated IQ [others: *t*_(9)_ = 2.76, *p* = 0.02; schizophrenia: *t*_(13)_ = 0.97, *p* = 0.35] and WRAT-R reading [others: *t*_(9)_ = 4.44, *p* = 0.002; schizophrenia: *t*_(13)_ = 0.61, *p* = 0.56].

**Figure 5:**
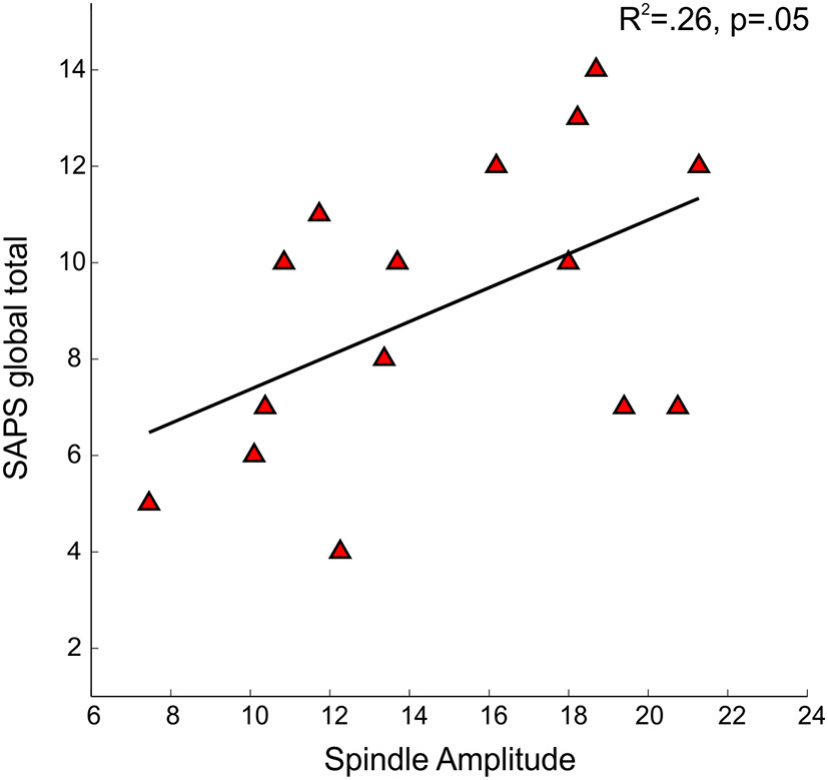
**Regressions of Scale for the Assessment of Positive Symptoms (SAPS) global total severity scores on spindle amplitude in early course schizophrenia patients**.

No significant relations between spindle density or amplitude with symptom rating scores or GAF were observed. Because we and another group previously found relations between reduced spindle amplitude (or “integrated spindle activity,” which is influenced by amplitude) and increased severity of positive symptoms in chronic, medicated schizophrenia patients (Ferrarelli et al., 2010; Wamsley et al., 2012), we examined the schizophrenia group alone and found a significant relation in the opposite direction: increased amplitude of individual spindles correlated with increased severity of positive symptoms [*t*_(13)_ = 2.21, *p* = 0.05] (Figure 5).

#### Control analyses

In addition to showing lower spindle density and amplitude than both healthy controls and psychotic patients with other diagnoses (trend for amplitude), the sleep of schizophrenia patients was also more disrupted. Sleep efficiency (a general measure of sleep quality), however, did not significantly correlate with spindle density or amplitude in schizophrenia, healthy controls or other psychotic patients, suggesting that sleep disruption is unlikely to account for the spindle deficits. Spindle density and amplitude correlated with multiple measures of cognition in the pooled group of early course patients. Sleep efficiency also correlated with several cognitive measures (Table 3), but notably not with estimated premorbid verbal IQ from the Ammons Quick Test or WRAT-R single word reading, which is also an estimate of premorbid verbal IQ.

### First-degree relatives of schizophrenia patients

#### Sleep quality, architecture, and spectral characteristics (Table 4 presents sleep data)

Compared with controls, relatives showed significantly worse sleep quality as indicated by increased WASO and reduced sleep efficiency. Sleep architecture was also disrupted in relatives who showed a greater percentage of time in lighter sleep (Stages 1 and 2) and trends toward lower percentages of slow wave and REM sleep. During Stage 2 sleep, relatives showed significant power reductions in all frequency bands except delta. To control for this shift in global power, we calculated sigma power relative to the EEG power baseline for each group, computed as the best fit to the 9–10 and 15–16 Hz data (Figure 1B). Sigma power (12–15 Hz) in relatives was only 25% of that seen in healthy controls.

**Table 4:** **Sleep data in relatives and controls**.

	**Relatives *n* = 19**	**Controls *n* = 12**	***t***	***p***
**SLEEP QUALITY**				
TST (min)	477 ± 61	511 ± 63	−1.36	0.19
WASO (min)	14 ± 14	5 ± 3	2.29	0.03^*^
Sleep efficiency %	93 ± 3	96 ± 2	−2.21	0.04^*^
**SLEEP ARCHITECTURE**				
Stage 1 %	4 ± 2	2 ± 1	2.01	0.05^*^
Stage 2 %	52 ± 7	45 ± 11	2.02	0.05^*^
SWS %	21 ± 8	27 ± 10	−1.78	0.09
REM %	23 ± 4	26 ± 4	−1.70	0.10
**SPECTRAL POWER DURING STAGE 2 SLEEP (μV^2^/HZ)**				
Slow (0.5–1 Hz)	10.6 ± 3.8	15.2 ± 6.3	−2.55	0.02^*^
Delta (1–4 Hz)	2.39 ± 0.97	3.04 ± 1.37	−1.54	0.13
Theta (4–8 Hz)	0.33 ± 0.17	0.49 ± 0.21	−2.38	0.02^*^
Alpha (8–12 Hz)	0.10 ± 0.05	0.17 ± 0.06	−3.29	0.003^*^
Sigma (12–15 Hz)	0.19 ± 0.10	0.44 ± 0.27	−3.60	0.001^*^
Beta (15–30 Hz)	0.008 ± 0.004	0.012 ± 0.004	−2.34	0.03^*^
**SPINDLE MEASURES**				
Spindle number	377 ± 89	399 ± 148	−0.52	0.61
Spindle density/min	1.52 ± 0.29	1.72 ± 0.33	−1.76	0.09
**CHARACTERISTICS OF INDIVIDUAL SPINDLES**				
Amplitude (μV)	18.2 ± 4.7	24.4 ± 6.8	−3.01	0.005^*^
Frequency (Hz)	12.9 ± 0.6	13.1 ± 0.8	−0.64	0.52
Duration (s)	0.844 ± 0.057	0.857 ± 0.049	−0.61	0.54
Sigma power(μV^2^/Hz)	0.555 ± 0.379	1.283 ± 0.847	−3.28	0.003^*^

Means ± SD; TST, total sleep time; WASO, wake after sleep onset; SWS, slow wave sleep; REM, rapid eye movement sleep.

* *Significant at p ≤ 0.05*.

#### Sleep spindle parameters

Relatives showed significantly reduced amplitude and sigma power of individual spindles, as well as a trend toward reduced spindle density. Relatives with and without psychiatric diagnoses did not differ in spindle density [*t*_(1, 17)_ = 1.16, *p* = 0.26] or amplitude [*t*_(1, 17)_ = 1.16, *p* = 0.26].

#### Spindle density and amplitude in relation to cognitive function and symptom ratings

Spindle density correlated with WCST perseverative errors in controls [*t*_(10)_ = 3.07, *p* = 0.01] but not relatives [*t*_(18)_ = 0.08, *p* = 0.94] and not in the pooled data of controls and relatives (Table 5). For the combined groups, spindle amplitude significantly correlated with GAF (*tdf* = 2.72, *p* = 0.01), but neither group alone showed this relation and the plot suggested it was due to group differences in both parameters. Spindle amplitude significantly correlated with IQ Table 5, (Figure 3) and showed a trend level relation with premorbid adjustment. These relations did not differ by group. None of the psychosis proneness ratings correlated with spindle density or amplitude in either the pooled group data or in either group alone.

**Table 5:** **Regressions of cognitive and symptom measures on spindle parameters in relatives and their controls**.

		**Group**		**Spindle × group**		**Spindle**	
		***t***	***p***	***t***	***p***	***t***	***p***
CPT	Density	0.68	0.50	−0.97	0.34	−1.13	0.27
Verbal	Amplitude	−0.28	0.78	−0.05	0.96	−0.20	0.84
CPT	Density	0.73	0.47	−1.02	0.32	0.50	0.62
Visual	Amplitude	−0.94	0.36	0.34	0.74	−0.13	0.90
WCST	Density	−1.78	0.09	2.01	0.06	−1.29	0.21
Pers err	Amplitude	0.61	0.55	−0.32	0.75	−1.34	0.19
IQ estimate	Density	−0.50	0.62	0.07	0.94	0.63	0.54
	Amplitude	0.44	0.66	−0.79	0.44	3.80	0.0007^*^
GAF	Density	−2.28	0.03^*^	0.58	0.57	1.26	0.22
	Amplitude	−1.63	0.12	−0.40	0.69	2.72	0.01^*^
PAS	Density	1.59	0.12	−1.09	0.28	−0.67	0.51
	Amplitude	0.99	0.33	−0.39	0.70	−1.80	0.08
Magical	Density	0.13	0.90	0.24	0.81	−0.23	0.82
Ideation	Amplitude	0.47	0.64	0.01	0.99	−0.78	0.44
Perceptual	Density	0.95	0.35	−0.64	0.53	−0.51	0.62
Aberration	Amplitude	1.40	0.17	−1.12	0.27	−1.69	0.10
Social	Density	−0.17	0.87	0.19	0.85	−2.01	0.07
Anhedonia	Amplitude	0.15	0.89	−0.01	0.99	−1.62	0.13

*Spindle refers to spindle parameter, density, or amplitude. Continuous Performance Test (CPT)—Identical Pairs version; WCST, Wisconsin Card Sort Test perseverative errors; GAF, Global Assessment of Functioning; PAS, Premorbid Adjustment Scale; Chapman Scales of Magical Ideation, Perceptual Aberration, and Social Anhedonia*.

* Significant at *p* ≤ 0.05.

#### Control analyses

Like the early course patients with schizophrenia, relatives showed reduced spindle density (trend) and amplitude relative to healthy controls, but their sleep quality was also more disrupted. Sleep efficiency, however, did not significantly correlate with spindle density or amplitude in relatives or their healthy controls, suggesting that the sleep disruption is unlikely to account for the spindle deficits. Nor did sleep efficiency correlate significantly with cognitive measures in relatives, controls, or the pooled group data.

## Discussion

The present study provides the first demonstration that both young first-degree relatives of patients with schizophrenia and antipsychotic-naïve patients early in the course of schizophrenia show reduced sleep spindle activity. In contrast, early course psychotic patients with other diagnoses showed normal spindle activity. These findings indicate that the spindle deficit, which was previously reported in chronic, medicated patients with schizophrenia (Ferrarelli et al., 2007, 2010; Manoach et al., 2010; Seeck-Hirschner et al., 2011; Wamsley et al., 2012), is not due to antipsychotic medications, is not a product of chronic illness and is not a general feature of psychosis. Moreover, consistent with growing evidence that links sleep spindles to a range of cognitive functions including intellectual ability in healthy individuals (Fogel and Smith, 2011), the present study found that sleep spindle activity correlated with multiple cognitive measures including estimates of verbal IQ in young healthy controls, early course psychotic patients, and young relatives of schizophrenia patients. Thus, spindle activity was related to cognitive function regardless of diagnosis. Together with prior work documenting a spindle deficit in chronic, medicated patients with schizophrenia that correlates with sleep-dependent memory consolidation (Wamsley et al., 2012), the present findings are consistent with the hypothesis that the spindle deficit is an endophenotype of schizophrenia that predates the onset of schizophrenia, is present throughout its course and affects cognitive function. Although suggestive, our findings are correlative and it is not possible to draw strong conclusions about causal relationships between spindles and cognitive function.

Recent work suggests sleep spindle activity as a potential target for the remediation of cognitive deficits in schizophrenia. Eszopiclone—a non-benzodiazapine sedative hypnotic that acts on the TRN, which generates sleep spindles (Jia et al., 2009)—significantly increased spindle activity compared with placebo in a small sample of chronic medicated schizophrenia patients (Wamsley et al., 2013). While its effect on sleep-dependent memory consolidation was not significant, only the eszopiclone group showed significant overnight improvement on the motor sequence task (Walker et al., 2002). Moreover, in the combined eszopiclone and placebo groups, spindle density predicted this overnight consolidation. These findings raise the possibility that spindle deficits can be effectively treated and that treatment may remediate cognitive deficits. This body of work, identifying abnormal sleep spindles as a potentially treatable candidate endophenotype of schizophrenia that is related to cognitive deficits, opens new avenues for research aimed at understanding, treating, and preventing schizophrenia.

The sleep spindle deficit in schizophrenia implicates dysfunction of thalamocortical circuitry. Sleep spindles are generated in the TRN (Guillery and Harting, 2003) and reduced spindle activity may reflect TRN and/or cortical dysfunction. There is evidence of TRN abnormalities in schizophrenia (Smith et al., 2001) and of reduced thalamic volume in antipsychotic-naïve first-episode schizophrenia (Gilbert et al., 2001). The TRN is comprised entirely of GABAergic neurons (Houser et al., 1980) that primarily inhibit glutamatergic thalamic neurons that project to the cortex. Cortical neurons, in turn, send glutamatergic inputs back to N-methyl-D-aspartate acid (NMDA) receptors on TRN neurons. Thus, spindles are mediated by a thalamocortical feedback loop that is regulated by both GABAergic and NMDA-receptor mediated glutamatergic neurotransmission (Jacobsen et al., 2001), which are implicated in current models of schizophrenia. In schizophrenia there is evidence of GABA deficits (Thompson et al., 2009) and abnormal expression of NMDA receptors and glutamate transporters in the thalamus (Ibrahim et al., 2000; Smith et al., 2001).

The correlations of spindle activity with IQ in the present samples are similar to what has been reported for healthy individuals in prior work (Fogel and Smith, 2011). Sleep spindles have been linked to a range of cognitive abilities in healthy individuals, particularly to the sleep-dependent consolidation of both procedural (Walker et al., 2002; Fogel and Smith, 2006; Nishida and Walker, 2007; Peters et al., 2008; Rasch et al., 2008; Tamaki et al., 2008) and declarative (Clemens et al., 2005, 2006; Schabus et al., 2008) memory. Converging evidence suggests that neocortical slow oscillations temporally group thalamocortical sleep spindles with hippocampal ripples thus enabling the redistribution of recently encoded memories from temporary hippocampal to long-term neocortical storage sites (Molle and Born, 2011). The coherent expression of spindles across wide areas of cortex could support the synchronous “reactivation” of recent memory traces across cortical regions (Buzsaki, 1998; O'Neill et al., 2010). In addition to reduced spindle activity, we previously found less coherent spindle activity across the cortex in chronic medicated schizophrenia (Wamsley et al., 2012). This may reflect dysfunction in thalamocortical circuits that could interfere with sleep-dependent memory processing preventing the simultaneous reactivation of memory components stored across visual, spatial, emotional, and goal-representation networks, resulting in the fragmentation of memories and cognition.

Consistent with this, in addition to its relations with estimates of premorbid verbal IQ (the Ammons Quick Test and WRAT-R Reading), sleep spindles correlated with multiple measures of cognitive performance. Sleep efficiency, a general measure of sleep quality, also correlated with cognitive measures in early course patients, but not in relatives, and it was not significantly correlated with IQ estimates or with spindle density or amplitude. This may reflect that while generalized sleep disruption affects the performance of many effortful and attentionally-demanding tasks (Van Dongen et al., 2003), the performance of tasks that primarily tap crystallized knowledge specifically relates to spindles.

Unlike chronic, medicated patients with schizophrenia in whom the sleep spindle reduction was found to be specific [i.e., with the exception of increased sleep onset latency in two studies (Ferrarelli et al., 2007, 2010), it occurred in the context of normal sleep quality, architecture, and other spectral characteristics of sleep (Manoach et al., 2010; Wamsley et al., 2012)] in both the early course schizophrenia patients and the relatives of schizophrenia patients, sleep was more generally disrupted. Early course patients with other psychotic disorders also showed disrupted sleep relative to controls, but the schizophrenia patients showed greater disruption as indicated by trends toward poorer sleep quality and significantly lower theta power during Stage 2 sleep. But the most compelling difference between schizophrenia patients and those with other psychotic disorders was the significantly reduced spindle activity including spindle density, sigma power and individual spindle duration and amplitude (trend). While schizophrenia patients significantly differed from healthy controls on multiple measures of spindle activity, those with other psychoses did not differ on any. In addition, as sleep efficiency was not significantly correlated with spindle density or amplitude in any group, a general sleep disruption is unlikely to fully account for the spindle deficits observed in schizophrenia patients or in young non-psychotic first-degree relatives. These findings suggest that sleep is disrupted in early course psychotic patients, but only those with schizophrenia show a spindle deficit. Not only was spindle density reduced, but schizophrenia patients also showed abnormal morphology of individual spindles (reduced amplitude and a trend to shorter duration) consistent with some (Ferrarelli et al., 2007, 2010) but not all (Wamsley et al., 2012) studies of chronic medicated patients.

A surprising observation was that positive symptoms were *positively* correlated with spindle amplitude in early course antipsychotic-naïve schizophrenia patients. This contrasts with the negative correlations previously observed in chronic medicated schizophrenia patients (Ferrarelli et al., 2010; Wamsley et al., 2012). This may reflect that the pathophysiological underpinnings of positive symptoms differ in these two populations. In chronic medicated patients, residual positive symptoms have not fully responded to standard dopamine blocking medications and may therefore arise from non-dopaminergic mechanisms such as GABA or NMDA hypofunction (Demjaha et al., 2014), which may also contribute to spindle deficits. In contrast, positive symptoms in early untreated schizophrenia typically respond well to antipsychotics and may reflect dopamine hyperactivity (Keshavan, 1999). These correlations suggest that, in addition to their putative role in cognition, sleep spindles may be related to the expression of schizophrenia symptoms, though the mechanisms of these relations are unknown. Spindle parameters did not correlate with measures of psychosis proneness in the combined group of relatives and their controls, or in the relatives alone.

There are several important limitations of the present study. First, we note that two prior studies of antipsychotic-naïve patients with schizophrenia did not show reduced spindle density during Stage 2 sleep. As in the present study, the sample sizes were relatively small *n* = 11 (Poulin et al., 2003) and *n* = 8 (Forest et al., 2007). Unlike the present study, the spindles were hand counted. This is unlikely to be the source of the discrepancy since the wavelet-based spindle counting algorithm used for the present study was previously validated against both hand-counted spindles and 12–15 Hz sigma power in both healthy individuals and patients with schizophrenia (Wamsley et al., 2012) and outperformed other available automated spindle detectors by most closely approximating expert consensus spindle counts (Warby et al., 2014). Given this discrepancy it will be important to replicate our findings in larger samples. The small sample sizes of the present study also left us underpowered for some analyses including those involving more complex models that could adjust for the effects of sleep efficiency or IQ on group differences in spindles. As this was an archival study, we were limited to available data and lacked information such as whether the time of day of cognitive and other functional measures was standardized across participants and groups. Because we were missing cognitive and some demographic measures for early course controls we also do not know whether they were well-matched to the early course patients on important demographic features such as parental socioeconomic status. This is a potential confound since the heritability of IQ varies as a function of parental socioeconomic status (e.g., Turkheimer et al., 2003) and IQ correlates with sleep spindles (e.g., Fogel and Smith, 2011). We do know, however, that the early course schizophrenia patients did not differ from other psychotic patients in age, sex, estimated IQ, positive, or negative symptom severity, or on a global functional assessment, yet only the schizophrenia patients showed a spindle deficit.

The group of young relatives had lower parental socioeconomic status than their controls. This may reflect socioeconomic slippage of the parents as a consequence of schizophrenia. The relatives also had lower estimated IQs, worse global function and more magical ideation and perceptual aberration, which may all be reflections of genetic vulnerability to schizophrenia and/or the psychosocial effects of having a first-degree family member with schizophrenia. Given these group differences, we cannot exclude the possibility that rather than reflecting genetic vulnerability to schizophrenia, the spindle deficit in relatives reflects differences in other factors such as IQ.

The present findings raise a number of important questions. Is reduced sleep spindle activity a genetic risk factor that predicts psychosis in high-risk individuals and in the prodromal phase? And, if so, would treating the spindle deficit improve cognition and/or reduce the probability of conversion to frank psychosis? And does the sleep spindle deficit help to illuminate the pathophysiology of pre-morbid stages of schizophrenia? Our findings implicate abnormal function in thalamocortical circuitry even before the onset of illness, which is consistent with a recent report of reduced volume of the thalamus bilaterally that correlated with sleep disturbance in adolescents at ultra high risk for psychosis (Lunsford-Avery et al., 2013). In chronic patients, would treating the spindle deficit improve cognition and symptoms and thereby reduce the risk of relapse?

These questions highlight important directions for future research. Sleep studies are non-invasive and the potential to remediate abnormal sleep for the prevention and treatment of schizophrenia should be examined. The detection of reduced spindle activity as a risk marker for conversion to schizophrenia in high-risk individuals and during the prodromal period would allow treatment of this deficit. In schizophrenia patients, treatment of the spindle deficit could potentially reduce the clinical, neurocognitive, and functional consequences of illness. In summary, we propose sleep spindles as a potential novel endophenotype and target for research and treatment development.

### Conflict of interest statement

Dr. David Kupfer has the following disclosures: Consultant to the American Psychiatric Association (as Chair of the DSM-5 Task Force); holds joint ownership of copyright for the Pittsburgh Sleep Quality Index (PSQI); received honorarium for manuscript submission to Medicographia (Servier); he is a member of the Valdoxan Advisory Board of Servier International; he is a stockholder in AliphCom; and he and his spouse, Dr. Ellen Frank are stockholders in Psychiatric Assessments, Inc.

## References

[B1] Ambrosius U. Lietzenmaier S. Wehrle R. Wichniak A. Kalus S. Winkelmann J. (2008). Heritability of sleep electroencephalogram. Biol. Psychiatry 64, 344–348 10.1016/j.biopsych.2008.03.002 18405882

[B2] American Psychiatric Association. (2000). Diagnostic and Statistical Manual of Mental Disorders, (4th Edn., text revision). Washington, DC: Author

[B3] Andersen R. (2008). Modern Methods for Robust Regression. Series on Quantitative Applications in the Social Sciences. Thousand Oaks, CA: SAGE Publications, 7–152

[B4] Andreasen N. C. (1983). Scale for the Assessment of Negative Symptoms (SANS). Iowa City, IA: University of Iowa

[B5] Andreasen N. C. (1990). Methods for assessing positive and negative symptoms. Mod. Probl. Pharmacopsychiatry 24, 73–88 2336066 10.1159/000418013

[B6] Benca R. M. (1996). Sleep in psychiatric disorders. Neurol. Clin. 14, 739–764 10.1016/S0733-8619(05)70283-8 8923493

[B7] Benson K. L. (2006). Sleep in schizophrenia: impairments, correlates, and treatment. Psychiatr. Clin. North Am. 29, 1033–1045; abstract: ix–x. 10.1016/j.psc.2006.08.002 17118280

[B8] Berg E. A. (1948). A simple objective technique for measuring flexibility in thinking. J. Gen. Psychol. 39, 15–22 10.1080/00221309.1948.9918159 18889466

[B9] Breslau N. Roth T. Rosenthal L. Andreski P. (1996). Sleep disturbance and psychiatric disorders: a longitudinal epidemiological study of young adults. Biol. Psychiatry 39, 411–418 10.1016/0006-3223(95)00188-3 8679786

[B10] Buzsaki G. (1998). Memory consolidation during sleep: a neurophysiological perspective. J. Sleep Res. 7(Suppl. 1), 17–23 10.1046/j.1365-2869.7.s1.3.x 9682189

[B11] Cannon-Spoor H. E. Potkin S. G. Wyatt R. J. (1982). Measurement of premorbid adjustment in chronic schizophrenia. Schizophr. Bull. 8, 470–484 10.1093/schbul/8.3.470 7134891

[B12] Chapman L. J. Chapman J. P. Raulin M. L. (1978). Body-image aberration in Schizophrenia. J. Abnorm. Psychol. 87, 399–407 10.1037/0021-843X.87.4.399 681612

[B13] Chouinard S. Poulin J. Stip E. Godbout R. (2004). Sleep in untreated patients with schizophrenia: a meta-analysis. Schizophr. Bull. 30, 957–967 10.1093/oxfordjournals.schbul.a007145 15954201

[B14] Clemens Z. Fabo D. Halasz P. (2005). Overnight verbal memory retention correlates with the number of sleep spindles. Neuroscience 132, 529–535 10.1016/j.neuroscience.2005.01.011 15802203

[B15] Clemens Z. Fabo D. Halasz P. (2006). Twenty-four hours retention of visuospatial memory correlates with the number of parietal sleep spindles. Neurosci. Lett. 403, 52–56 10.1016/j.neulet.2006.04.035 16714084

[B16] Cornblatt B. A. Risch N. J. Faris G. Friedman D. Erlenmeyer-Kimling L. (1988). The Continuous Performance Test, identical pairs version (CPT-IP): I. New findings about sustained attention in normal families. Psychiatry Res. 26, 223–238 10.1016/0165-1781(88)90076-5 3237915

[B17] De Gennaro L. Marzano C. Fratello F. Moroni F. Pellicciari M. C. Ferlazzo F. (2008). The electroencephalographic fingerprint of sleep is genetically determined: a twin study. Ann. Neurol. 64, 455–460 10.1002/ana.21434 18688819

[B18] Delis D. C. Freeland J. Kramer J. H. Kaplan E. (1988). Integrating clinical assessment with cognitive neuroscience: construct validation of the California Verbal Learning Test. J. Consult. Clin. Psychol. 56, 123–130 10.1037/0022-006X.56.1.123 3346437

[B19] Demjaha A. Egerton A. Murray R. M. Kapur S. Howes O. D. Stone J. M. (2014). Antipsychotic treatment resistance in schizophrenia associated with elevated glutamate levels but normal dopamine function. Biol. Psychiatry 75, e11–e13 10.1016/j.biopsych.2013.06.011 23890739

[B20] Dencker S. J. Malm U. Lepp M. (1986). Schizophrenic relapse after drug withdrawal is predictable. Acta Psychiatr. Scand. 73, 181–185 10.1111/j.1600-0447.1986.tb10584.x 3705994

[B21] Eckblad M. Chapman L. J. (1983). Magical ideation as an indicator of schizotypy. J. Consult. Clin. Psychol. 51, 215–225 10.1037/0022-006X.51.2.215 6841765

[B22] Ferrarelli F. Huber R. Peterson M. J. Massimini M. Murphy M. Riedner B. A. (2007). Reduced sleep spindle activity in schizophrenia patients. Am. J. Psychiatry 164, 483–492 10.1176/appi.ajp.164.3.483 17329474

[B23] Ferrarelli F. Peterson M. J. Sarasso S. Riedner B. A. Murphy M. J. Benca R. M. (2010). Thalamic dysfunction in schizophrenia suggested by whole-night deficits in slow and fast spindles. Am. J. Psychiatry 167, 1339–1348 10.1176/appi.ajp.2010.09121731 20843876 PMC2970761

[B24] First M. B. Spitzer R. L. Gibbon M. Williams J. B. W. (1997). Structured Clinical Interview for DSM-IV Axis I Disorders, Research Version, Patient Edition with Psychotic Screen (SCID-I/P W/PSY SCREEN). New York, NY: Biometrics Research, New York State Psychiatric Institute

[B25] First M. B. Spitzer R. L. Gibbon M. Williams J. B. W. (2002). Structured Clinical Interview for DSM-IV-TR Axis I Disorders, Research Version, Nonpatient Edition. New York, NY: Biometrics Research, New York State Psychiatric Institute

[B26] Fogel S. M. Smith C. T. (2006). Learning-dependent changes in sleep spindles and Stage 2 sleep. J. Sleep Res. 15, 250–255 10.1111/j.1365-2869.2006.00522.x 16911026

[B27] Fogel S. M. Smith C. T. (2011). The function of the sleep spindle: a physiological index of intelligence and a mechanism for sleep-dependent memory consolidation. Neurosci. Biobehav. Rev. 35, 1154–1165 10.1016/j.neubiorev.2010.12.003 21167865

[B28] Ford D. E. Kamerow D. B. (1989). Epidemiologic study of sleep disturbances and psychiatric disorders. An opportunity for prevention? JAMA 262, 1479–1484 10.1001/jama.1989.03430110069030 2769898

[B29] Forest G. Poulin J. Daoust A. M. Lussier I. Stip E. Godbout R. (2007). Attention and non-REM sleep in neuroleptic-naive persons with schizophrenia and control participants. Psychiatry Res. 149, 33–40 10.1016/j.psychres.2005.11.005 17141330

[B30] Germain A. Buysse D. J. Nofzinger E. (2008). Sleep-specific mechanisms underlying posttraumatic stress disorder: integrative review and neurobiological hypotheses. Sleep Med. Rev. 12, 185–195 10.1016/j.smrv.2007.09.003 17997114 PMC2490669

[B31] Gilbert A. R. Rosenberg D. R. Harenski K. Spencer S. Sweeney J. A. Keshavan M. S. (2001). Thalamic volumes in patients with first-episode schizophrenia. Am. J. Psychiatry 158, 618–624 10.1176/appi.ajp.158.4.618 11282698

[B32] Goder R. Fritzer G. Gottwald B. Lippmann B. Seeck-Hirschner M. Serafin I. (2008). Effects of olanzapine on slow wave sleep, sleep spindles and sleep-related memory consolidation in schizophrenia. Pharmacopsychiatry 41, 92–99 10.1055/s-2007-1004592 18484550

[B33] Gottesman I. I. Gould T. D. (2003). The endophenotype concept in psychiatry: etymology and strategic intentions. Am. J. Psychiatry 160, 636–645 10.1176/appi.ajp.160.4.636 12668349

[B34] Guillery R. W. Harting J. K. (2003). Structure and connections of the thalamic reticular nucleus: advancing views over half a century. J. Comp. Neurol. 463, 360–371 10.1002/cne.10738 12836172

[B35] Hiatt J. F. Floyd T. C. Katz P. H. Feinberg I. (1985). Further evidence of abnormal non-rapid-eye-movement sleep in schizophrenia. Arch. Gen. Psychiatry 42, 797–802 10.1001/archpsyc.1985.01790310059007 4015324

[B36] Hollingshead A. B. (1965). Two Factor Index of Social Position. New Haven, CT: Yale University Press

[B37] Houser C. R. Vaughn J. E. Barber R. P. Roberts E. (1980). GABA neurons are the major cell type of the nucleus reticularis thalami. Brain Res. 200, 341–354 10.1016/0006-8993(80)90925-7 7417821

[B38] Huang Y. S. Guilleminault C. Li H. Y. Yang C. M. Wu Y. Y. Chen N. H. (2007). Attention-deficit/hyperactivity disorder with obstructive sleep apnea: a treatment outcome study. Sleep Med. 8, 18–30 10.1016/j.sleep.2006.05.016 17157069

[B39] Ibrahim H. M. Hogg A. J. Jr. Healy D. J. Haroutunian V. Davis K. L. Meador-Woodruff J. H. (2000). Ionotropic glutamate receptor binding and subunit mRNA expression in thalamic nuclei in schizophrenia. Am. J. Psychiatry 157, 1811–1823 10.1176/appi.ajp.157.11.1811 11058479

[B40] Jacobsen R. B. Ulrich D. Huguenard J. R. (2001). GABA(B) and NMDA receptors contribute to spindle-like oscillations in rat thalamus *in vitro*. J. Neurophysiol. 86, 1365–1375 11535683 10.1152/jn.2001.86.3.1365

[B41] Jastak S. Wilkinson G. (1984). The Wide Range Acheivement Test-Revised: Administration Manual. Wilmington, DE: Jastak Associates

[B42] Jia F. Goldstein P. A. Harrison N. L. (2009). The modulation of synaptic GABA(A) receptors in the thalamus by eszopiclone and zolpidem. J. Pharmacol. Exp. Ther. 328, 1000–1006 10.1124/jpet.108.146084 19033556

[B43] Kahn-Greene E. T. Killgore D. B. Kamimori G. H. Balkin T. J. Killgore W. D. (2007). The effects of sleep deprivation on symptoms of psychopathology in healthy adults. Sleep Med. 8, 215–221 10.1016/j.sleep.2006.08.007 17368979

[B44] Keshavan M. S. (1999). Development, disease and degeneration in schizophrenia: a unitary pathophysiological model. J. Psychiatr. Res. 33, 513–521 10.1016/S0022-3956(99)00033-3 10628528

[B45] Keshavan M. S. Diwadkar V. A. Montrose D. M. Stanley J. A. Pettegrew J. W. (2004). Premorbid characterization in schizophrenia: the Pittsburgh High Risk Study. World Psychiatry 3, 163–168 16633488 PMC1414704

[B46] Keshavan M. S. Montrose D. M. Miewald J. M. Jindal R. D. (2011). Sleep correlates of cognition in early course psychotic disorders. Schizophr. Res. 131, 231–234 10.1016/j.schres.2011.05.027 21724373 PMC3217835

[B47] Kraepelin E. (1919). Dementia Praecox and Paraphrenia. Edinburgh: E.S. Livingston

[B48] Lieberman J. A. Stroup T. S. McEvoy J. P. Swartz M. S. Rosenheck R. A. Perkins D. O. (2005). Effectiveness of antipsychotic drugs in patients with chronic schizophrenia. N. Engl. J. Med. 353, 1209–1223 10.1056/NEJMoa051688 16172203

[B49] Lunsford-Avery J. R. Orr J. M. Gupta T. Pelletier-Baldelli A. Dean D. J. Smith Watts A. K. (2013). Sleep dysfunction and thalamic abnormalities in adolescents at ultra high-risk for psychosis. Schizophr. Res. 151, 148–153 10.1016/j.schres.2013.09.015 24094679 PMC3855888

[B50] Manoach D. S. Thakkar K. N. Stroynowski E. Ely A. McKinley S. K. Wamsley E. (2010). Reduced overnight consolidation of procedural learning in chronic medicated schizophrenia is related to specific sleep stages. J. Psychiatr. Res. 44, 112–120 10.1016/j.jpsychires.2009.06.011 19665729 PMC2813320

[B51] Miller T. J. Zipursky R. B. Perkins D. Addington J. Woods S. W. Hawkins K. A. (2003). The PRIME North America randomized double-blind clinical trial of olanzapine versus placebo in patients at risk of being prodromally symptomatic for psychosis. II. Baseline characteristics of the “prodromal” sample. Schizophr. Res. 61, 19–30 10.1016/S0920-9964(02)00440-1 12648732

[B52] Mishlove M. Chapman L. J. (1985). Social anhedonia in the prediction of psychosis proneness. J. Abnorm. Psychol. 94, 384–396 10.1037/0021-843X.94.3.384 4031235

[B53] Molle M. Born J. (2011). Slow oscillations orchestrating fast oscillations and memory consolidation. Prog. Brain Res. 193, 93–110 10.1016/B978-0-444-53839-0.00007-7 21854958

[B54] Nishida M. Walker M. P. (2007). Daytime naps, motor memory consolidation and regionally specific sleep spindles. PLoS ONE 2:e341 10.1371/journal.pone.0000341 17406665 PMC1828623

[B55] O'Neill J. Pleydell-Bouverie B. Dupret D. Csicsvari J. (2010). Play it again: reactivation of waking experience and memory. Trends Neurosci. 33, 220–229 10.1016/j.tins.2010.01.006 20207025

[B56] Orvaschel H. Puig-Antich J. (1987). Schedule for Affective Disorders and Schizophrenia for School-Age Children: Epidemiologic 4th version. Ft. Lauderdale, FL: Nova University, Center for Psychological Study

[B57] Otto W. McMenemy R. A. (1965). An appraisal of the ammons quick test in a remedial reading program. J. Educ. Meas. 2, 193–198 10.1111/j.1745-3984.1965.tb00415.x

[B58] Peters K. R. Ray L. Smith V. Smith C. (2008). Changes in the density of stage 2 sleep spindles following motor learning in young and older adults. J. Sleep Res. 17, 23–33 10.1111/j.1365-2869.2008.00634.x 18275552

[B59] Poulin J. Daoust A. M. Forest G. Stip E. Godbout R. (2003). Sleep architecture and its clinical correlates in first episode and neuroleptic-naive patients with schizophrenia. Schizophr. Res. 62, 147–153 10.1016/S0920-9964(02)00346-8 12765755

[B60] Rasch B. Pommer J. Diekelmann S. Born J. (2008). Pharmacological REM sleep suppression paradoxically improves rather than impairs skill memory. Nat. Neurosci. 12, 396–397 18836440 10.1038/nn.2206

[B61] Rechtschaffen A. Kales A. (1968). A Manual Standardized Terminology, Techniques and Scoring System for Sleep Stages of Human Subjects. Bethesda, MD: National Institute of Neurological Diseases and Blindness, Neurological Information Network

[B62] Reitan R. M. (1958). Validity of the Trail Making Test as an indication of organic brain damage. Percept. Mot. Skills 8, 251–276 10.2466/pms.1958.8.3.271

[B63] Rosanova M. Ulrich D. (2005). Pattern-specific associative long-term potentiation induced by a sleep spindle-related spike train. J. Neurosci. 25, 9398–9405 10.1523/JNEUROSCI.2149-05.2005 16221848 PMC6725710

[B64] Sateia M. J. (2009). Update on sleep and psychiatric disorders. Chest 135, 1370–1379 10.1378/chest.08-1834 19420207

[B65] Schabus M. Hoedlmoser K. Pecherstorfer T. Anderer P. Gruber G. Parapatics S. (2008). Interindividual sleep spindle differences and their relation to learning-related enhancements. Brain Res. 1191, 127–135 10.1016/j.brainres.2007.10.106 18164280 PMC2855382

[B66] Seeck-Hirschner M. Baier P. C. Sever S. Buschbacher A. Aldenhoff J. B. Goder R. (2011). Effects of daytime naps on procedural and declarative memory in patients with schizophrenia. J. Psychiatr. Res. 44, 42–47 10.1016/j.jpsychires.2009.05.008 19559446

[B67] Smith R. E. Haroutunian V. Davis K. L. Meador-Woodruff J. H. (2001). Expression of excitatory amino acid transporter transcripts in the thalamus of subjects with schizophrenia. Am. J. Psychiatry 158, 1393–1399 10.1176/appi.ajp.158.9.1393 11532723

[B68] Tamaki M. Matsuoka T. Nittono H. Hori T. (2008). Fast sleep spindle (13-15 hz) activity correlates with sleep-dependent improvement in visuomotor performance. Sleep 31, 204–211 18274267 10.1093/sleep/31.2.204 PMC2225572

[B69] Thompson M. Weickert C. S. Wyatt E. Webster M. J. (2009). Decreased glutamic acid decarboxylase(67) mRNA expression in multiple brain areas of patients with schizophrenia and mood disorders. J. Psychiatr. Res. 43, 970–977 10.1016/j.jpsychires.2009.02.005 19321177

[B70] Turek F. W. (2005). Insomnia and depression: if it looks and walks like a duck. Sleep 28, 1362–1363 16335326

[B71] Turkheimer E. Haley A. Waldron M. D'Onofrio B. Gottesman I. I. (2003). Socioeconomic status modifies heritability of IQ in young children. Psychol. Sci. 14, 623–628 10.1046/j.0956-7976.2003.psci_1475.x 14629696

[B72] Tyler D. B. (1955). Psychological changes during experimental sleep deprivation. Dis. Nerv. Syst. 16, 293–299 13261891

[B73] Van Cauter E. Linkowski P. Kerkhofs M. Hubain P. L'Hermite-Baleriaux M. Leclercq R. (1991). Circadian and sleep-related endocrine rhythms in schizophrenia. Arch. Gen. Psychiatry 48, 348–356 1848971 10.1001/archpsyc.1991.01810280064009

[B74] Van Dongen H. P. Maislin G. Mullington J. M. Dinges D. F. (2003). The cumulative cost of additional wakefulness: dose-response effects on neurobehavioral functions and sleep physiology from chronic sleep restriction and total sleep deprivation. Sleep 26, 117–126 12683469 10.1093/sleep/26.2.117

[B75] Walker M. P. Brakefield T. Morgan A. Hobson J. A. Stickgold R. (2002). Practice with sleep makes perfect: sleep-dependent motor skill learning. Neuron 35, 205–211 10.1016/S0896-6273(02)00746-8 12123620

[B76] Wamsley E. J. Shinn A. K. Tucker M. A. Ono K. E. McKinley S. K. Ely A. V. (2013). The effects of eszopiclone on sleep spindles and memory consolidation in schizophrenia: a randomized placebo-controlled trial. Sleep 36, 1369–1376 10.5665/sleep.2968 23997371 PMC3738047

[B77] Wamsley E. Tucker M. A. Shinn A. K. Ono K. E. McKinley S. Ely A. V. (2012). Reduced sleep spindles and spindle coherence in schizophrenia: mechanisms of impaired memory consolidation? Biol. Psychiatry 71, 154–161 10.1016/j.biopsych.2011.08.008 21967958 PMC3561714

[B78] Warby S. C. Wendt S. L. Welinder P. Munk E. G. Carrillo O. Sorensen H. B. (2014). Sleep-spindle detection: crowdsourcing and evaluating performance of experts, non-experts and automated methods. Nat. Methods 11, 385–392 10.1038/nmeth.2855 24562424 PMC3972193

[B79] Wechsler D. (1981). The Wechsler Adult Intelligence Scale-Revised. New York, NY: Psychological Corporation

[B80] Wehr T. A. Sack D. A. Rosenthal N. E. (1987). Sleep reduction as a final common pathway in the genesis of mania. Am. J. Psychiatry 144, 201–204 3812788 10.1176/ajp.144.2.201

[B81] Werk C. M. Harbour V. L. Chapman C. A. (2005). Induction of long-term potentiation leads to increased reliability of evoked neocortical spindles *in vivo*. Neuroscience 131, 793–800 10.1016/j.neuroscience.2004.12.020 15749334

[B82] Wright J. B. (1993). Mania following sleep deprivation. Br. J. Psychiatry 163, 679–680 10.1192/bjp.163.5.679 8298841

