# Supplementary material for: Sleep spindle deficits in antipsychotic-naïve early course schizophrenia and in non-psychotic first-degree relatives
Source: Front Hum Neurosci. 2014 Oct 7;8:762. doi: 10.3389/fnhum.2014.00762 (PMC4188028; doi:10.3389/fnhum.2014.00762)
Supplement: Supplementary file 1 [file Table1.DOCX]

Supplemental Table 1. Neuropsychological data. Means ±SD, SZ: early course schizophrenia patients; Other: early course patients with other psychotic disorders; Wisconsin Card Sort Test perseverative errors (WCST Pers Error), Trail Making Tests Parts A and B, Block Design scaled score, the Wide Range Achievement Test-Revised, Reading standard score ([WRAT-R](#_ENREF_31) ) and the California Verbal Learning Test standard score for total immediate word recall (CVLT), Continuous Performance Test – Identical Pairs version (Cont. Perf. Verbal, Cont. Perf. Visual).

|  | **SZ**  **n=15** | **Other**  **n=11** | ***t*** | ***p*** |
| --- | --- | --- | --- | --- |
| **WCST Pers Error** | 21±14 | 15±12 | 1.15 | .26 |
| **Trails A (sec)** | 32±17 | 26±9 | 1.21 | .24 |
| **Trails B (sec)** | 88±34 | 62±49 | 1.61 | .12 |
| **Block Design SS** | 10±2.9 | 11.5±2.8 | -.84 | .41 |
| **WRAT-R Std** | 100±13 | 101±19 | -.15 | .88 |
| **CVLT Std** | 24±20 | 32±13 | -1.24 | .23 |
|  | **Relatives**  **n=19** | **Controls**  **n=12** | ***t*** | ***p*** |
| **WCST Pers Error** | 17±8 | 13±6 | 1.69 | .11 |
| **Cont. Perf. Verbal** | 1.0±.70 | 1.3±.56 | -1.21 | .24 |
| **Cont. Perf. Visual** | .94±.73 | 1.4±.93 | -1.43 | .17 |

Supplemental Table 2. Clincal characteristics of study samples. Means ±SD, SZ: early course schizophrenia patients; Other: early course patients with other psychotic disorders; SANS: Scale for the Assessment of Negative Symptoms; SAPS: Scale for the Assessment of Positive Symptoms; GAF: Global Assessment of Functioning Scale; Chapman Scales of Magical Ideation; Perceptual Aberration; and Social Anhedonia; Premorbid Adjustment Scale (PAS).

|  | SZ  n=15 | Other  n=11 | *t* | *p* |
| --- | --- | --- | --- | --- |
| SANS global total | 14±3 | 12±3 | 1.22 | .23 |
| SAPS global total | 9±3 | 8±5 | 0.72 | .48 |
| GAF | 35±11 | 39±9 | 1.05 | .30 |
|  | Relatives  n=19 | Controls  n=12 |  | *p* |
| Mag. Ideation | .19±.16 | .08±.09 | 2.11 | .04* |
| Percept. Aber. | .11±.10 | .04±.06 | 2.09 | .05* |
| Social Anhed. | .20±.14 | .13±.20 | 0.79 | .44 |
| GAF | 69±9 | 95±3 | 9.26 | <.001* |
| PAS | .25±.16 | .09±.09 | 3.16 | .004* |
